# Sexual size dimorphism in anurans: roles of mating system and habitat types

**DOI:** 10.1186/1742-9994-10-65

**Published:** 2013-11-07

**Authors:** Wen Bo Liao, Yu Zeng, Jian Dong Yang

**Affiliations:** 1 Key Laboratory of Southwest China Wildlife Resources Conservation (Ministry of Education), China West Normal University, Nanchong, Sichuan 637009, China; 2 Colleges of Animal Science, Sichuan Agricultural University, Ya’an 625014, China; 3 China Three Gorges Corporation, Beijing 100038, China

**Keywords:** Anuran, Habitat type, Mating system, Phylogenetic comparative analysis, Sexual size dimorphism

## Abstract

**Background:**

Sexual size dimorphism (SSD) is widespread and variable among animals. Sexual selection, fecundity selection and ecological divergence between males and females are the major evolutionary forces of SSD. However, the influences of mating system and habitat types on SSD have received little attention. Here, using phylogenetic comparative methods, we at first examine the hypotheses to that mating system (intensity of sexual selection) and habitat types affect significantly variation in SSD in anurans (39 species and 18 genera).

**Results:**

Our data set encompass 39 species with female-biased SSD. We provide evidence that the effects of mating system and habitat types on SSD were non-significant across species, also when the analyses were phylogenetically corrected.

**Conclusions:**

Contrast to the hypotheses, our findings suggest that mating system and habitat types do not play an important role in shaping macro-evolutionary patterns of SSD in anurans. Mating system and habitat types cannot explain the variation in SSD when correcting for phylogenetic effects.

## Introduction

Sexual size dimorphism (SSD) is widespread and variable among animals [1]. In some groups (e.g. birds, lizards and most mammals), males are bigger than females, whereas in other groups (fishes and anurans) females are bigger than males [2]. Some key hypotheses are provided for explaining the evolution and maintenance of SSD, although their explanatory power remains controversial [3-5]. It is now widely agreed that sexual selection in favor of large males to improve intra-sexual combat success and fecundity selection for large females to increase reproductive output are the major evolutionary forces of SSD in many organisms [1]. Beyond the two hypotheses, ecological divergence between the sexes due to intraspecific competition has been proposed to explain evolution of SSD [6].

SSD is often used as an indicator of the intensity of sexual selection in animals [7]. Mating system is associated with the intensity of sexual selection [1]. The idea is that promiscuous species where females mate with more than one male have more intensive competition than monogamous species where females mate with only one male. Sexual selection hypothesis predicts that the intensity of selection promotes variation of SSD among species through intra-sexual competition or inter-sexual mate choice favoring large size in one sex [1,7]. Consequently, mating system can mediate evolution of SSD. Moreover, habitats types have been proposed to affect SSD due to energy constraints and predators. For example, small male size in aquatic habitats resulting from selection to reduce energy expenditure in mate searching shows a female-biased SSD, whereas large male size in terrestrial habitats results from predation pressure, mate searching needs, or desiccation avoidance, showing a mixed SSD [6,8,9]. For frogs it appears that aquatic and arboreal species have usually smaller variation in SSD than terrestrial species [10].

Anurans inhabit a wide range of habitats (i.e. wetlands, grasslands, steams, trees and ponds) and exhibit a remarkable diversity of mating system (i.e. social polyandry and social monogamy) that is unique among vertebrates [10,11]. If the selection hypotheses are valid, we expect mating system and habitat types having effects on SSD in anurans. Comparative studies on anuran SSD have been shown that differences in the age structure between the sexes in breeding populations can explain variation in SSD [12-15]. Moreover, several attempts have been made to explain SSD as a consequence of sexual selection, fecundity selection and life-history traits in anurans [16,17]. However, the influences of mating system and habitat types on SSD in anurans have received little attention. Here, we at first examine the hypotheses to that mating system and habitat types significantly influence on variation of SSD.

## Results

**Figure 1:**
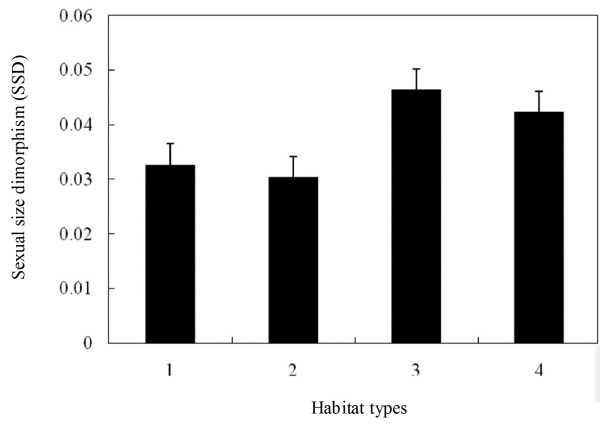
**Sexual dimorphism in body size [mean ± SD log**_**10 **_**(female size)-log**_**10 **_**(male size)] in relation to mating system among 39 anuran species.**

**Figure 2:**
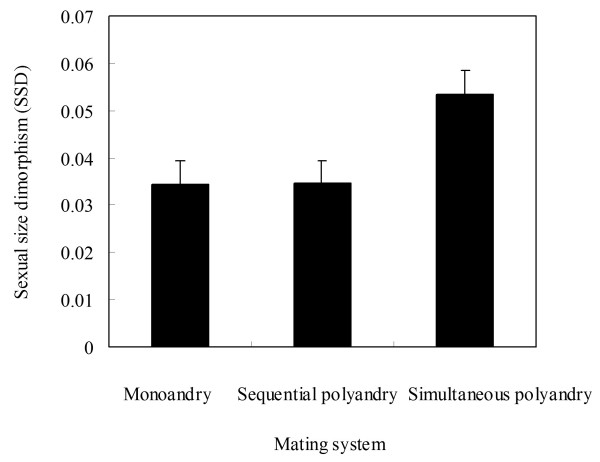
**Sexual dimorphism in body size [mean ± SD log**_**10 **_**(female size)-log**_**10 **_**(male size)] in relation to habitat types among 39 anuran species.** 1 = Terrestrial - mostly occur on ground, forage in ground in various conditions, 2 = Semiaquatic - not entirely aquatic, usually living or growing in or near water, 3 = Arboreal – mostly occur on trees, forage in trees and rarely come down to the ground, 4 = Aquatic – mostly occur on water, forage in water in stream.

Thirty-nine species were characterised by female-biased SSD. The GLM revealed that the mean SVL significantly differed between the sexes (*F*_1, 77_ = 3.059, *P* = 0.043) and terrestrial, arboreal, semiaquatic and aquatic habitats (*F*_3, 77_ = 2.756, *P* = 0.050), but did not differ among sequential polyandry, simultaneous polyandry and monoandry (*F*_2, 77_ = 0.641, *P* = 0.530). However, there were also non-significant mating systems*sex interaction (*F*_2, 77_ = 0.036, *P* = 0.964) and habitats *sex interaction (*F*_3, 77_ = 0.125, *P* = 0.945), revealing that the degree – but not direction (females always the larger sex) – of SSD did not differ among mating system and habitats (Figure 1 and 2).

We tested for the effects of mating system and habitats on SSD contrasts using generalized least squares. We found the mean SVL contrasts significantly differed between the sexes (*F*_1, 75_ = 3.182, *P* = 0.041) and habitats (*F*_3, 75_ = 4.342, *P* = 0.020), but did not differ among mating system (*F*_2, 75_ = 0.038, *P* = 0.963). However, the non-significant interactions between mating system and sex (*F*_2, 75_ = 0.017, *P* = 0.983) and between habitats and sex (*F*_3, 75_ = 0.042, *P* = 0.997) revealed the evidence that intensity of sexual selection and habitats were not associated with variation of SSD contrasts.

## Discussion

Ninety percent anuran species is characterised by female-biased SSD, and 10% by male-biased SSD [2]. In our study, female-biased SSD is the predominant pattern. Sexual selection supports the idea that males engaging in physical combat with one another may select for large males when such males are more successful in gaining access to mates [1]. However, male–male competition prevails in some anurans where a female-biased SSD is common [16]. This means that selection on large females should be stronger than selection on large males due to the expression of a given SSD depending on relative dominance of competing selective forces [17]. Such a source of selection in favor of large females is considered to be the size-dependent fecundity advantage, which can drive the evolution of female body size and consequently leads to the evolution of female-biased SSD [16]. Moreover, females begin breeding later, live longer but grow more slowly than males, resulting in female-larger patterns of SSD in anuran lineages. In particular, SSD is increasingly biased towards females across species when the duration of growth in females is longer than males [18].

Mating system is often used as an indicator of the intensity of sexual selection [1]. Our results show that SSD is not affected by mating system, suggesting that anurans with intense sexual selection do not exhibit a range of dimorphisms. This pattern is contradictory to the well-established concept that male-male competition (sexual selection) drives to increase male body size and results in male-biased SSD [1]. Several potential causes can explain why intensity of sexual selection does not promote evolution of SSD in anurans [1,11,15,19]. Firstly, mating success is positively correlated with male body size in some species, but not in others. Secondly, an advantage for small males relates to post-copulatory sexual selection through sperm competition. The idea is that small males have low chances of obtaining females through female choice or male–male competition, so they should instead disproportionately invest in mate search and sperm competition, where they are assumed to have relatively better chances. Thirdly, the occurrence of alternative mating tactics in anurans reduces the selection pressure on male body size. Consequently, small males may gain fitness by using alternative mating tactics. Finally, the extreme diversity of life-history traits and their plasticity may mask the potential contribution of male-male competition to body size evolution.

Mating system mediates SSD in birds, such that under polyandry, sexual selection on females results in a SSD pattern opposite to Rensch’s rule while selection on males results in a SSD pattern consistent with Rensch’s rule [20]. Evidence in support of this idea has been obtained from Rensch’s rule is driven by a correlated evolutionary response in one sex to stronger size selection in the other sex. However, contrast to the hypothesis, we find that mating system in anurans cannot mediate SSD, such that sexual selection on males results in a SSD pattern inconsistent with Rensch’s rule and it’s reverse [13]. The possible reason is that fecundity selection for large females balances out sexual selection on large males.

Anurans are a diverse group of vertebrates renowned for variable life-history traits, which include mainly terrestrial, arboreal, semiaquatic and aquatic habitats [10]. For example, terrestrial species exhibit male combat, and males are as large or larger than females. For aquatic and arboreal species that exhibit female choice due to male mating calls, males are smaller than females. Consequently, terrestrial species should show larger variation in SSD than aquatic and arboreal species [10,21]. However, our results suggest that in anurans the regimes of natural selection imposed by habitat types alone may not have exerted a significant impact on body size in either of the sexes. This finding supports the assumption that males and females are ecologically or phenotypically equivalent which may provide an incomplete or even mistaken picture of the process of body size diversification [22]. In conclusion, variation of SSD in anurans cannot be explained by mating system and habitat types. It is the result of a variety of selective forces, including sexual selection, fecundity selection, life-history and ecological factors.

In this study, methodological aspects give reason to view our results with caution. At first, the phylogenetic tree we present appears to be a simple dendrogram showing just branching pattern, i.e. its branch spans do not represent time or relative amount of character change. PGLM methods make explicit use of information contained in branch lengths. However, we cannot obtain a tree with branch lengths, so we use GLM to test variation of SSD contrasts which may outcome potential problems and biases which may occur as a consequence of ignoring this information. At second, correlations across species should be regarded cautiously based on the fact that species’ data points cannot be assumed to be statistically independent [23,24]. However, comparisons across species still result in meaningful analyses unless they need a cluster of points that share an immediate common ancestor Harvey and Pagel [25].

## Materials and methods

We obtained sex-specific demographic and morphological data on mean size across 39 species and 18 genera from the literatures (Additional file 1: Table S1). We calculated the mean values for the population as algebraic means for each year, weighted by sample size. Mean values for species were obtained as algebraic means of population values regardless of the sample size in cases where data were available for different population [12]. Following the method proposed by Roberts and Byrne [26], we used mating system as an imperfect surrogate of the intensity of sexual selection on a three-point scale: 1 = sequential polyandry where two or more males simultaneously releasing sperm or sequentially releasing sperm in a time frame that allows for the occurrence of sperm competition; 2 = simultaneous polyandry where a females mates with two or more males over the course of a breeding season by depositing part of a single clutch with each male or, multiple clutching; 3 = monoandry where a females mates with one male over the course of a breeding season by depositing part of a single clutch. Habitat types were classified on a four-point scale: 1 = Terrestrial - mostly occur on ground, forage in ground in various conditions, 2 = Semiaquatic - not entirely aquatic, usually living or growing in or near water, 3 = Arboreal – mostly occur on trees, forage in trees and rarely come down to the ground, 4 = Aquatic – mostly occur on water, forage in water in stream. Following the methods by Lovich and Gibbons [27], we calculated SSD as (log_10_ (female mean size)/log_10_ (male mean size)) - 1, arbitrarily set positive when females are larger and negative when males are larger. All animals used in this study were treated humanely and ethically following all applicable institutional Animal Care guidelines in China.

Comparative analyses of interspecific data may require phylogenetic control as closely related species share parts of their evolutionary history. Therefore, they cannot be considered independent data points for statistical analyses [24]. Phylogenetic analyses were based on generalized least squares, which is a powerful and comprehensive approach to the analysis of comparative data [28]. Generalized least squares is a modification of generalized linear models (GLMs) in which the phylogeny is used to specify the expected variance and covariance between species under an assumed. For our comparative analysis, we used an established phylogeny [29,30] (Additional file 2: Figure S1). We calculated mean size in both sexes for ancestral nodes as the algebraic mean of the two closest lower nodes [23]. Details of the general procedure for estimating the character values in the ancestors are presented in Felsenstein [24]. With 39 species at the tips of this reconstructed tree, 38 (39–1) body size of contrasts within each sex could be computed for pairs of nodes sharing an immediate common ancestor, and then re-scaled and analysed as suggested by Garland et al. [31]. Correct standardization and homogeneity of variance of standardized contrasts were confirmed using the method proposed by Purvis and Rambaut [32]. To conduct the effect of mating system and habitat types on variation in SSD, we conducted conventional non-phylogenetic GLMs separately on log (body size) as a dependent variable, habitats, mating system, sex and their interactions as fixed factors. We then used GLMs separately on log (body size) contrasts as a dependent variable, and mating system, habitats, sex and their interactions as fixed factors to test the effects of mating system and habitat types on SSD contrasts. All tests were conducted by using Type III sums of squares.

## Competing interests

The authors have declared that no competing interests exist.

## Authors’ contributions

WBL and YZ carried out the analyses and drafted the manuscript. WBL and JDY designed the study. All the authors read and approved the final manuscript.

## Supplementary Material

Additional file 1: Table S1 Species, sample size (females/males), mean body size in males and females of 39 species and references of published papers. Click here for file

Additional file 2: Figure S1 The phylogenetic tree of the 39 anuran species used in the comparative analysis following Frost et al. (2006) and Pyron and Wiens (2011). Click here for file
