# Supplementary material for: Sexual size dimorphism in anurans: roles of mating system and habitat types
Source: Front Zool. 2013 Nov 7;10:65. doi: 10.1186/1742-9994-10-65 (PMC3879228; doi:10.1186/1742-9994-10-65)
Supplement: Additional file 1: Table S1 — Species, sample size (females/males), mean body size in males and females of 39 species and references of published papers. [file 1742-9994-10-65-S1.doc]

Additional file 1: Table S1. Species, sample size (females/males), mean body size in males and females of 39 species and references of published papers.

| Species | Samplings  females/males | Females SVL | Males  SVL | Mating system | Habitat types | References |
| --- | --- | --- | --- | --- | --- | --- |
| *Bufo bufo* | 485/837 | 72.7 | 59.2 | Sequential polyandry | Terrestrial in various conditions | 13,14,40 |
| *Rana sylvatica* | 402/891 | 52.6 | 46.1 | Sequential polyandry | Semiaquatic in or near water | 15,16,17,40 |
| *Rana temporaria* | 141/227 | 75.5 | 69.7 | Sequential polyandry | Semiaquatic in or near water | 18,19,20,40 |
| *Mixophyes fasciolatus* | 56/107 | 75.2 | 64.9 | Monoandry | Aquatic in stream | 1,40 |
| *Mixophyes fleayi* | 9/40 | 51.8 | 49.2 | Monoandry | Aquatic in stream | 1,40 |
| *Pelophylax ridibunda* | 56/52 | 82.4 | 69.0 | Monoandry | Semiaquatic in or near water | 2,40 |
| *Pelophylax pleuraden* | 70/125 | 49.8 | 43.8 | Monoandry | Semiaquatic in or near water | 3,41,42 |
| *Pelophylax nigromaculata* | 148/209 | 76.6 | 67.0 | Monoandry | Semiaquatic in or near water | 4,5,6,41 |
| *Hylarana guentheri* | 79/86 | 75.1 | 67.8 | Monoandry | Semiaquatic in or near water | 7,41,42 |
| *Fejervarya limnocharis* | 65/172 | 46.4 | 40.0 | Monoandry | Semiaquatic in or near water | 8,41,42 |
| *Bufo americanus* | 37/34 | 71.5 | 64.3 | Monoandry | Terrestrial in various conditions | 9,40 |
| *Bufo cognatus* | 10/21 | 66.4 | 58.0 | Monoandry | Terrestrial in various conditions | 10,40 |
| *Bufo punctatus* | 8/25 | 59.2 | 52.0 | Monoandry | Terrestrial in various conditions | 10,40 |
| *Bufo alvarius* | 9/9 | 125.0 | 117.8 | Monoandry | Terrestrial in various conditions | 10,40 |
| *Bufo andrewsi* | 280/432 | 99.2 | 77.9 | Monoandry | Terrestrial in various conditions | 11,40 |
| *Bufo gargarizans* | 79/128 | 101.1 | 92.9 | Monoandry | Terrestrial in various conditions | 12,41,42 |
| *Nanorana parkeri* | 987/1355 | 42.5 | 37.4 | Monoandry | Semiaquatic in or near water | 21,41,42 |
| *Alytes obstetricans* | 31/59 | 49.7 | 41.7 | Monoandry | Semiaquatic in or near water | 22,40 |
| *Alytes cisternasii* | 13/74 | 38.6 | 35.8 | Monoandry | Semiaquatic in or near water | 22,40 |
| *Rana omeimontis* | 64/63 | 69.7 | 62.6 | Monoandry | Semiaquatic in or near water | 23,42 |
| *Rana chensinensis* | 270/513 | 46.5 | 41.8 | Monoandry | Semiaquatic in or near water | 24,25,26,41,42 |
| *Rana catesbeiana* |  | 113.2 | 105.3 | Monoandry | Semiaquatic in or near water | 27,40 |
| *Rana muscosa* | 74/44 | 63.6 | 56.1 | Monoandry | Semiaquatic in or near water | 28,40 |
| *Pelobates fuscus* | 29/87 | 53.9 | 45.9 | Monoandry | Semiaquatic in or near water | 29,40 |
| *Xenopus laevis* | 64/51 | 63.3 | 59.9 | Monoandry | Semiaquatic in or near water | 30,40 |
| *Litoria chloris* |  | 64.1 | 58.3 | Monoandry | Arboreal in trees | 1,40 |
| *Litoria lesueuri* |  | 59.9 | 39.2 | Monoandry | Arboreal in trees | 1,40 |
| *Hyla arborea* | 49/94 | 44.4 | 43.1 | Monoandry | Arboreal in trees | 31,40 |
| *Hyla crucifer* | 59/184 | 28.4 | 26.5 | Monoandry | Arboreal in trees | 32,40 |
| *Mantidactylus microtympanum* | 26/33 | 94.1 | 74.7 | Monoandry | Arboreal in trees | 35,41,42 |
| *Phyllomedusa boliviana* |  | 74.6 | 67.9 | Monoandry | Arboreal in trees | 38,40 |
| *Odorrana grahami* | 93/108 | 76.3 | 69.1 | Monoandry | Aquatic in stream | 12,41,42 |
| *Amolops lifanensis* | 30/20 | 68.9 | 50.7 | Monoandry | Aquatic in stream | 39,41,42 |
| *Amolops mantzorum* | 112/170 | 68.5 | 53.7 | Monoandry | Aquatic in stream | 40,41,42 |
| *Hyla annectans* | 33/64 | 39.5 | 32.5 | Simultaneous polyandry | Arboreal in trees | 33,40 |
| *Rhacophorus megacephalus* | 21/108 | 69.3 | 51.6 | Simultaneous polyandry | Arboreal in trees | 12,41,42 |
| *Rhacophorus omeimontis* | 51/203 | 75.5 | 62.6 | Simultaneous polyandry | Arboreal in trees | 34,42 |
| *Chiromantis xerampelina* | 30/34 | 76.1 | 63.2 | Simultaneous polyandry | Arboreal in trees | 36,40 |
| *Phyllomedusa burmeisteri* | 13/13 | 76.7 | 63.4 | Simultaneous polyandry | Arboreal in trees | 37,40 |

References

1. Morrison FC: *Altitudinal Variation in the Life History of Anurans in Southeast Queensland*. Australia: Unpublished Ph.D., Griffith University, Bundall, Queensland; 2001.

2. Kyriakopoulou-Sklavounou P, Stylianou P, Tsiora A: **A skeletochronological study of age, growth and longevity in a population of the frog *Rana ridibunda* from southern Europe**. *Zoology* 2008, **111**: 30–36.

# 3. Lou SL, Jin L, Liu YH, Mi ZP, Tao G, Tang YM, Liao WB: Altitudinal variation in age and body size in Yunnan Pond Frog (*Pelophylax pleuraden*). *Zool Sci* 2012, 29: 493–498.

4. Liao WB, Zhou CQ, Yang ZS, Lu X: **Age, size and growth in two populations of the dark-spotted frog *Rana nigromaculata* at different altitudes in southwestern China**. *Herpetol J* 2010, **20**: 77–82.

5. Mao M, Huang Y, Mi ZP, Liu YH, Zhou CQ: Skeletochronological study of age, longevity and growth in a population of *Rana nigromaculata* (Amphibia: Anura) in Sichuan, China. *Asian Herpetol Res* 2012, **3(3)**: 258–264.

6. Khonsue W, Matsui M, Hirai T, Misawa Y: **A comparison of age structures in two populations of a pond frog *Rana nigromaculata* (Amphibia: Anura)**. *Zool Sci* 2001, **18**: 597–603.

7. Li C, Liao WB, Yang ZS, Zhou CQ: **A skeletochronological estimation of age structure in a population of the Guenther’s frog, *Hylarana guentheri*, from western China**. *Acta Herpetol* 2010, **5**: 1–11.

8. Liao WB, Lu X, Shen YW, Hu JC: **Age structure and body size of two populations of the rice frog *Rana limnocharis* from different altitudes**. *Ital J Zool* 2011, **78**: 215–221.

9. Acker PM, Kruse KC, Krehbiel, EB: **Aging *Bufo americanus* by skeletochronology**. *J Herpetol* 1986, **20**: 570–574.

10. Sullivan BK, Fernandez PJ: **Breeding activity, estimated age-structure, and growth in sonoran desert anurans**. *Herpetologica* 1999, **55**: 334–343.

11. Liao WB, Lu X: **Adult body size = *f* (initial size + growth rate × age): explaining the proximate cause of Bergman’s cline in a toad along altitudinal gradients**. *Evol Ecol* 2012, **26**: 579–590.

12. Liao WB, Zeng, Y, Zhou CQ, Jehle R: **Sexual size dimorphism in anurans fails to obey Rensch's rule**. *Front Zool* 2013, **10**: 10.

13. Cvetković D, Tomašević N, Ficetola GF, Crnobrnja-Isailović J, Miaud C: **Bergmann’s rule in amphibians: combining demographic and ecological parameters to explain body size variation among populations in the common toad *Bufo bufo***. [*J Zool Syst Evol Res*](http://www.wiley.com/bw/submit.asp?ref=0947-5745) 2009, **47**: 171–180.

14. Hemelaar ASM: **Age, growth and other population characteristics of *Bufo bufo* from different latitudes and altitudes**. *J Herpetol* 1988, **22**: 369–388.

15. Bastien H, Leclair Jr R: **Aging wood frogs (*Rana sylvatica*) by skeletochronology**. *J Herpetol* 1992, **26**: 222–225.

16. Berven KA: **The genetic basis of altitudinal variation in the wood frog, *Rana sylvatica*. I. An experimental analysis of life-history traits**. *Evolution* 1982, **36**: 962–983.

17. Sagor ES, Ouellet M, Barten E, Green DM: Skeletochronology and geographic variation in age structure in the wood frog, *Rana sylvatica*. *J Herpetol* 1998, **32**: 469–474.

18. Guarino FM, Erismis UC: **Age determination and growth by skeletochronology of *Rana holtzi*, an endemic frog from Turkey**. *Ital J Zoo*l 2008, **73**: 237–242.

19. Ryser J: **Determination of growth and maturation in the common frog, *Rana temporaria*, by skeletochronology**. *J Zool* 1988, **216**: 673–685.

20. Miaud C, Guyétant R, Elmberg J: **Variations in life history traits in the common frog *Rana temporaria* (Amphibia: Anura): a literature review and new data from the French Alps**. *J Zool* 1999, **249**: 61–73.

21. Ma XY, Tong LN, Lu X: **Variation of body size, age structure and growth of a temperate frog, *Rana chensinensis*, over an elevational gradient in northern China***. Amphibia-Reptilia*. 2009, **30**: 111–117.

22. Márquez R, Esteban M, Castanet J: **Sexual size dimorphism in midwife toads *Alytes obstetricans* and *A. cisternasii***. *J Herpetol* 1997, **31**: 52–59.

23. Liu WC, Liu YH, Huang Y, Mi ZP, Li C: Skeletochronological study on age structure of a Chinese endemic frog (*Rana omeimontis*). *Asian Herpetol Res* 2012, **3(3)**: 252–257.

24. Lu X, Li B, Liang JJ: 2006 **Comparative demography of a temperate anuran, *Rana chensinensis*, along a relatively fine altitudinal gradient**. [*Can J Zool*](http://proquest.umi.com/pqdweb?RQT=318&pmid=36147&TS=1199187314&clientId=26439&VInst=PROD&VName=PQD&VType=PQD) **84**: 1789–1795.

25. Ma XY, Tong LN, Lu X: **Variation of body size, age structure and growth of a temperate frog, *Rana chensinensis*, over an elevational gradient in northern China*.*** *Amphibia-Reptilia* 2009, 30: 111–117.

26. Chen BY, Liao WB, Mi ZP: **Body size and age of the China Wood Frog (*Rana chensinensis*) in northeastern China**. *N-W J Zool* 2011, **7**: 236–242.

27. Shirose LJ, Brooks RJ, Barta JR, Desser SS: **Intersexual differences in growth, mortality, and size at maturity in bullfrogs in central Ontario**. *Can J Zool* 1993, **71**: 2363–2369.

28. Matthews KR, Miaud C: **A skeletochronological study of the age structure, growth, and longevity of the mountain yellow-legged frog, *Rana muscosa*, in the Sierra Nevada, California**. *Copeia* 2007, **2007**: 986–993.

29. Eggert C, Guyétant R: **Age structure of a spadefoot toad *Pelobates fuscus* (Pelobatidae) population**. *Copeia* 1999, **1999**: 1127–1130.

30. Measey GJ: **Growth and ageing of feral *Xenopus laevis* (Daudin) in South Wales, UK**. *J Zool* 2001, **254**: 547–555.

31. Friedl TWP, Klump GM: **Some aspects of population biology in the European treefrog, *Hyla arborea***. *Herpetologica* 1997, **53**: 321–330.

32. Lykens DV, Forester DC: **Age structure in the spring peeper: do males advertise longevity**? *Herpetologica* 1987, **43**: 216–223.

33. Liao WB, Lu X: **Age structure and body size of the Chuanxi tree toad *Hyla annectans chuanxiensis* from two different elevations (China)**. *Zool Anz* 2010a, **248**: 255–263.

34. Liao WB, Lu X: **Variation in body size, age and growth in a subtropical treefrog (*Rhacophorus omeimontis*) along an altitudinal gradient in western China**. *Ethol Ecol Evol* 2011, **23**: 248–261.

35. Guarino FM, Andreone F, Angelini F: **Growth and longevity by skeletochronological analysis in *Mantidactylus* *microtympanum*, a rain-forest anuran from southern Madagascar**. *Copeia* 1998, **1998**: 194–198.

36. Byrne PG, Whiting MJ: **Simultaneous polyandry increases fertilization success in an African foam-nesting treefrog**. *Anim Beha* 2008, **76**: 1157–1164.

37. Abrunhosa PA, Woge H: **Breeding behavior of the leaf-frog *Phyllomedusa burmeisteri* (Anura: Hylidae)***.* *Amphibia-Reptilia* 2004, **25**: 125–135.

38. Vaira M: **Breeding biology of the leaf frog, *Phyllomedusa boliviana* (Anura, Hylidae)**. *Amphibia-Reptilia* 2001, **22**: 421–429.

39. Liu YH, Liao WB, Zhou CQ, Mi ZP, Mao M: **Age structure of *Amolops lifanensis****. J China West Norm Univ (Nat Sci)* 2011, **23**: 151–155.

40. Duellman WE, Trueb L: *Biology of Amphibians*. New York: McGraw-Hill; 1986.

41. Zhao EM, Adler K: *Herpetology of China*. Oxford: Society for the Study of Amphibians and Reptiles; 1993.

42. Fei L, Hu SQ, Ye CY, Huang YZ: *Fauna of Sinica.* [*Amphibia*](http://www.iciba.com/Amphibia)*, Volume II, Anuran*. Beijing: Science Press; 2009.
